# Aggregation-Induced Emission-Active Cyanostilbene-Based Liquid Crystals: Self-Assembly, Photophysical Property, and Multiresponsive Behavior

**DOI:** 10.3390/molecules29235811

**Published:** 2024-12-09

**Authors:** Bian Li, Junde Zhang, Juan Wang, Xiaofang Chen

**Affiliations:** Suzhou Key Laboratory of Macromolecular Design and Precision Synthesis, Jiangsu Key Laboratory of Advanced Functional Polymer Design and Application, State and Local Joint Engineering Laboratory for Novel Functional Polymeric Materials, College of Chemistry, Chemical Engineering and Materials Science, Soochow University, Suzhou 215123, China; bianli1995@163.com (B.L.); 20224209177@stu.suda.edu.cn (J.Z.); 18020222552@163.com (J.W.)

**Keywords:** cyanostilbene, liquid crystal, aggregation-induced emission, thermoresponsive, photoresponsive

## Abstract

Cyanostilbene (CS)-related conjugated groups can be considered as dual functional groups of AIEgen and mesogen to construct photoluminescent liquid crystals, and it is essential to study the relationship between their molecular structures and compound properties systematically. In this paper, we designed and synthesized linear and bent-shaped CS derivatives containing ester- and amide-connecting groups and different substituted numbers of alkoxy tails. Their phase behaviors and photophysical properties were investigated in depth. The bent-shaped compounds with the mono-substituted alkoxy tail exhibit a smectic C structure, and those containing two or three alkoxy tails possess a hexagonal columnar phase structure. The compounds exhibit aggregation-induced emission (AIE) properties in tetrahydrofuran (THF)/water mixtures. When the water fraction increases to a certain threshold, a dramatic increase in emission intensity and a red-shift in the fluorescence emission peak are detected. The emission peaks of the ester-type compounds in solid states are around 480 nm, and those of the amide-type compounds are extended to 590 nm, exhibiting versatile luminescent colors. Moreover, thermochromic and photochromic fluorescence-responsive properties are witnessed in these CS derivatives. This work provides a new strategy for the design and synthesis of fluorescent liquid crystalline materials with multiple response properties.

## 1. Introduction

Luminescent liquid crystals (LLCs), combining the ordered alignment of liquid crystals (LC) and luminescence properties, have gained much attention because of their potential applications in chemosensors, liquid crystal displays, polarized organic lasers, and information storage [1,2,3,4,5]. Different fluorescent chromophores have been explored to obtain efficient luminous LC compounds. The general molecule construction strategy is to combine the fluorescent group and mesogen. Since high luminescence should be kept condensed, molecular-aggregation-induced fluorescence quenching must be avoided, hindering the further development of LLCs. Recently, fluorophores that exhibit aggregation-induced emission (AIE) properties [6,7,8,9,10], called AIEgens, have been considered excellent fluorescent chromophore candidates in the molecular design of LLC systems.

Among various AIEgens, cyanostilbene (CS)-related groups are considered unique building blocks with AIEgen and mesogen characteristics [11,12,13,14,15,16,17,18,19]. The conjugated CS group exhibits a typical rod-like structure, which can be used as a primary unit to construct various LLCs instead of traditional LC molecules or complex supramolecular LC systems. To date, nematic (N) [20,21,22,23,24,25] and various smectic [22,26,27,28,29,30,31,32,33,34,35] phases have been formed in CS derivatives with end-attached mono-substituted alkoxy tails. Columnar phases (Col), such as Col_h_ [28,36,37,38,39,40,41,42,43,44,45,46,47,48,49,50,51,52], Col_sque_ [53,54], and Col_r_ [27,28,42,43,44,45,46,47,55,56,57,58,59], can be obtained in polycatenar LCs that contain multiple substituted alkoxy tails, as well as LC molecules with bent-shaped, disk-like, or star-like rigid cores. Cubic phases [40,60] can be found in CS-based amphiphilic LCs. To date, various CS-based molecular structures have been designed and synthesized and their LC behaviors have been investigated. Most of the reported work focuses on the design of conjugated structures and altering the length of alkoxy tails, while the effect of the number of substituted alkoxy tails [60] and linking groups [40] on LC behavior are rarely reported.

**Scheme 1:**
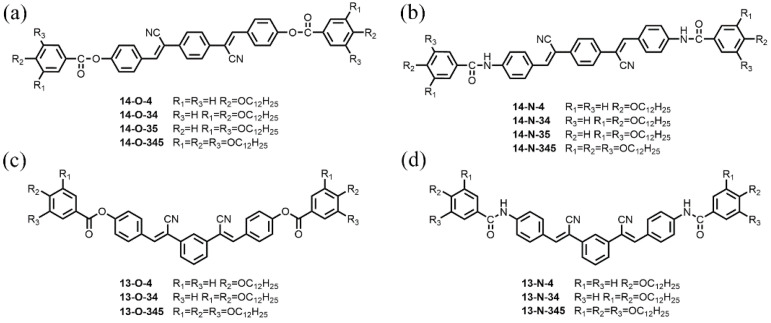
Chemical structures of (**a**) linear ester-type, (**b**) linear amide-type, (**c**) bent-shaped ester-type, and (**d**) bent-shaped amide-type compounds.

Furthermore, CS-related groups exhibit versatile responsive behavior and have been widely used to construct multiple stimulus-responsive materials. The luminescence intensity and wavelength can be readily modulated under various external stimuli, such as temperature [26,37], mechanical force [61], and light [16,62,63]. Current studies on AIE-active CS-based LCs mainly focus on investigating responsive properties and developing new functions [18,19,63,64]. It has been found in some cases that a small difference in CS-based molecular structure would induce a large difference in photophysical and responsive properties, especially in solid states [65,66,67]. So, it is rather challenging to comprehensively consider the molecular structural influence on LC’s behaviors, photophysical properties, and responsive properties during molecular design. Systematic studies on the relationship between the structure of molecules and the properties of compounds are increasingly important. Herein, we design a series of linear and bent-shaped CS derivatives containing an ester or amide bond in the rigid block and a different number of substituted alkoxy tails at both ends of the rigid core (Scheme 1). The differences in their chemical structures include the central benzene ring’s substitution positions, the linking groups, and the number of alkoxy tail chains. This study explores the influences of these structures on the phase behaviors and photophysical properties of CS derivatives, finding smectic and columnar phases in the system. The fluorescence properties can be readily tuned by varying the conjugated structure, linking groups, and alkoxy tails, achieving a wide emission wavelength range from 400 nm to 590 nm and thermochromic and photochromic fluorescence-responsive properties. These investigations can guide the development of novel LLC materials.

## 2. Results and Discussion

### 2.1. Synthesis and Characterization

**Figure 1:**
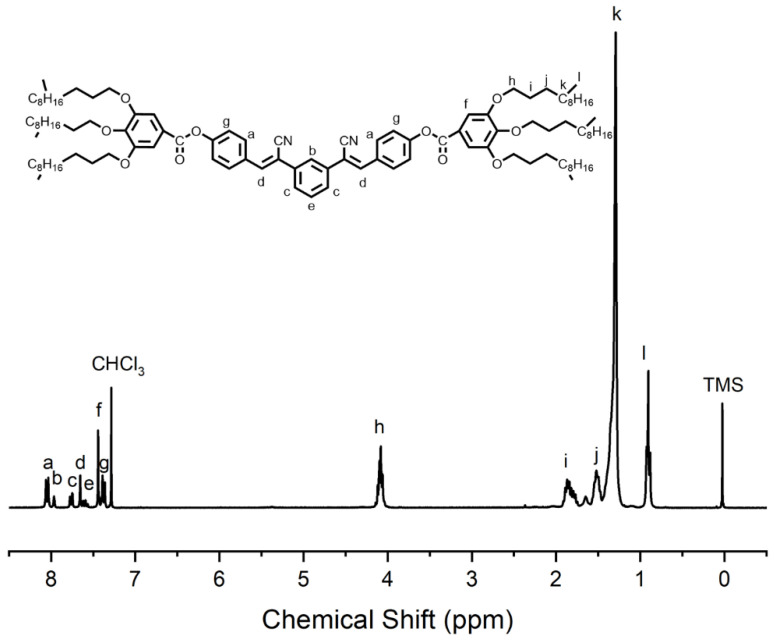
^1^H NMR spectrum of bent-shaped ester-type compound 13-O-345.

This study synthesized all compounds using the synthetic route, as shown in Scheme S1. Ester-type compounds were synthesized through etherification, Knoevenagal condensation, and esterification; amide-type compounds were synthesized via etherification, amidation, an oxidizing reaction, and Knoevenagal condensation. It has been found that some linear compounds, including 14-O-4, 14-N-4, 14-O-34, and 14-N-34, cannot be purified due to their poor solubility. Thus, the properties of these compounds were not studied here. The structure and purity of the other compounds were confirmed by hydrogen nuclear magnetic resonance (^1^H NMR) and mass spectrometry (MS). Figure 1 shows the ^1^H NMR spectrum of 13-O-345, an example of successfully synthesized linear and bent-shaped compounds. The thermal stability of the compounds was characterized by thermogravimetric analysis (TGA). The decomposition temperature was over 300 °C when the sample reached a weight loss of 5% under a nitrogen atmosphere. Therefore, the compounds’ phase behaviors were studied below this temperature.

### 2.2. Liquid Crystalline Behaviors

The phase behaviors of the compounds were studied by combining differential scanning calorimetry (DSC), polarized optical microscopy (POM), and small-angle X-ray scattering (SAXS). The phase transition temperatures and enthalpy data of the compounds are summarized in Table 1. Generally, amide-type compounds exhibit a much higher isotropic temperature (*T*_i_) than ester-type compounds due to the hydrogen bonding interaction of amide groups. In addition, bent-shaped compounds show a lower phase transition temperature than linear compounds, attributed to their less conjugated structures and weaker intermolecular interactions. *T*_i_ is also related to the number of substituted alkoxy tails. As shown in Table 1, mono-substituted 13-O-4 has the highest *T*_i_, and multi-substituted 13-O-345 manifests the lowest *T*_i_ due to the plasticization of flexible alkoxy tails. Among the linear compounds, 14-O-35 has a lower *T*_i_ than 14-O-345, while 14-N-35 has a higher *T*_i_ than 14-N-345. The former phenomenon is because the *meta* substituted tails tend to interrupt the ordered packing. The latter happens because the tri-substituted alkoxy tails has a larger volume fraction than the di-substituted one, which tends to decrease the intermolecular hydrogen bonding interactions in the linear imide compounds.

It can be seen from Table 1 that 14-O-35, 14-O-345, and 14-N-35 only show one phase transition during the heating or cooling process. POM observation confirms that this phase transition belongs to a crystal-to-isotropic transition, indicating no presence of mesophase in these compounds. 14-N-345 exhibits monotropic phase behavior. One phase transition peak at 185 °C is detected during heating, consistent with the isotropic temperature. When heating to the isotropic temperature, there is no mesophase under POM, but a crystal-to-liquid transition can be found. Two-phase transition peaks at 180 °C and 174 °C are present when cooling from the isotropic state, which can be further confirmed via POM observation. When cooling from the isotropic liquid state, fan-like textures can be found at 180 °C that quickly transform into crystals at 176 °C (Figure S1). Therefore, the LC phase structure is difficult to detect because of its narrow LC phase temperature range and easy crystallization. Thus, in this system, dicyanodistyrylbenzene-based rod-shaped compounds do not show a stable mesomorphic phase, which is due to the strong intermolecular interaction of the rod-like rigid cores. Changing the rigid core from a rod-shaped to a bent-shaped structure would partially reduce the strong π-π interaction from the conjugation structure, which may benefit the stabilization of mesophase formation.

**Figure 2:**
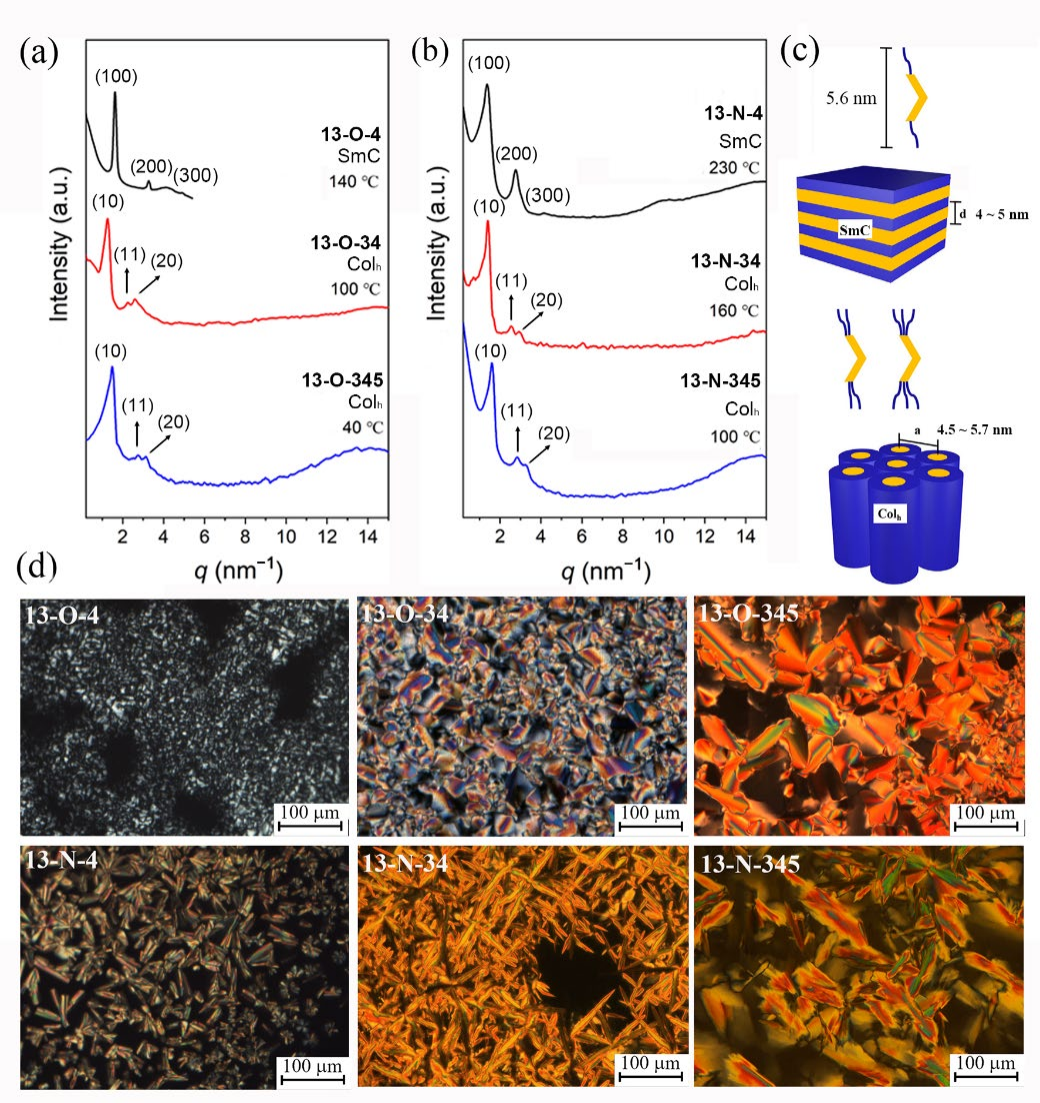
(**a**) SAXS profiles of 13-O-4 at 140 °C, 13-O-34 at 100 °C, and 13-O-345 at 40 °C; (**b**) SAXS profiles of 13-N-4 at 230 °C, 13-N-34 at 160 °C, and 13-N-345 at 100 °C; (**c**) schematic illustration of molecular packing in SmC and Col_h_ phases; (**d**) POM pictures of 13-O-4 at 160 °C, 13-O-34 at 105 °C, 13-O-345 at 40 °C, 13-N-4 at 220 °C, 13-N-34 at 150 °C, and 13-N-345 at 110 °C.

Unlike linear compounds, bent-shaped compounds can enter the LC state during heating and cooling cycles, presenting enantiotropic thermal behavior. SAXS was performed to determine the LC structures of the compounds. The SAXS profile of 13-O-4 at 140 °C (Figure 2a) shows three diffraction peaks at 1.64, 3.28, and 4.92 nm^−1^ with a *q* ratio of 1:2:3, indicating a layer-like smectic structure. The calculated layer thickness (*d*) is 3.8 nm. Note that the length (*L*) of the extended molecular structure with all-trans conformation of the alkoxy chain is around 5.6 nm, larger than the layer thickness. This indicates that the molecules are tilted within the layer, giving rise to a smectic C (SmC) structure. The corresponding POM picture shows a schlieren texture during cooling from the isotropic state (Figure 2b). When the number of substituted alkoxy tails is increased, the resulting compounds, 13-O-34 and 13-O-345, exhibit different phase structures. The SAXS profile of 13-O-34 (Figure 2a) shows three diffraction peaks at 1.28, 2.22, and 2.57 nm^−1^ with a *q* ratio of 1:3^1/2^:2, corresponding to the (10), (11), and (20) signals of the two-dimensional (2D) hexagonal columnar (Col_h_,) mesophase. The calculated lattice parameter is *a* = 5.7 nm, comparable to the molecular length. The SAXS profile of 13-O-345 (Figure 2a) also shows three diffraction peaks with the same *q* ratio as that of 13-O-34. A typical Col_h_ structure with a lattice parameter of *a* = 4.7 nm is confirmed (Figure 2a). The POM picture in Figure 2d shows the characteristic fan-like and dendritic textures of 13-O-34 and 13-O-345 growing from the isotropic state during cooling, consistent with the SAXS result. Considering the molecular structure, 13-O-34 and 13-O-345 first self-assemble into cylindrical aggregates with rigid cores and soft alkoxy tails as shells. Then, these columns are closely packed into the hexagonal lattice, as shown in Figure 2c.

The SAXS profile of the bent-shaped amide-type compound 13-N-4 taken at 230 °C (Figure 2b) indicates a smectic structure. POM shows the schlieren and broken focal conic textures growing (Figure 2d) from the isotropic state. The *d*-spacing is 4.6 nm, smaller than the fully extended molecular length, indicating the formation of the SmC structure with molecules tilting within the layer. 13-N-34 and 13-N-345, which contain multiple alkoxy tails, possess a 2D columnar phase. The SAXS profile of 13-N-34 reveals three diffraction peaks at 1.45, 2.53, and 2.92 nm^−1^ with a *q* ratio of 1:3^1/2^:2, indicating a characteristic Col_h_ structure (Figure 2b). Its lattice parameter is *a* = 5.0 nm. The SAXS profile of 13-N-345 also confirms a Col_h_ structure with a lattice parameter of *a* = 4.4 nm (Figure 2b). Both show characteristic fan-like and dendritic textures when cooling from the isotropic state (Figure 2d).

Based on the investigations above, a bent-shaped rigid core, instead of a linear core, is suitable for mesomorphic phase formation. The existence of amide groups can raise the phase transition temperature, and the number of substituted alkoxy tails can further modulate the phase structure.

### 2.3. Photophysical Properties

The photophysical properties of the compounds in solution were first studied via ultraviolet–visible spectroscopy (UV-vis) and photoluminescence spectroscopy (PL). The UV-vis absorption spectra of the compounds in dilute tetrahydrofuran (THF) solution are shown in Figure S2. The ester-type compounds with rod-like and bent-shaped structures exhibit maximum absorption peaks (*λ*_abs_) at 360 nm and 315 nm, respectively, indicating that the effective conjugation length is reduced from rod-like to bent-shaped compounds. The same trends can also be found in amide-type compounds with the absorption bands located at 390 nm and 360 nm for linear and bent-shaped conjugated ones, respectively. Furthermore, the *λ*_abs_ of amide-type compounds is larger than that of ester-type compounds, because the rigid amide group can extend the effective conjugation length.

**Figure 3:**
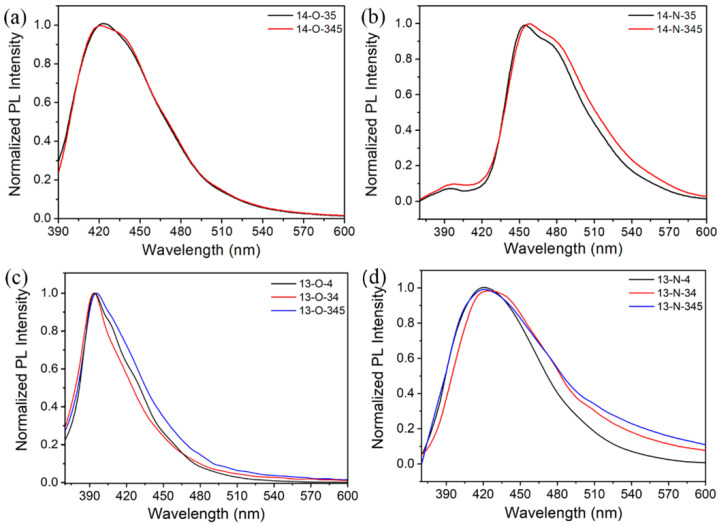
Normalized PL spectra of (**a**) 14-O-35 and 14-O-345; (**b**) 14-N-35 and 14-N-345; (**c**) 13-O-4, 13-O-34, and 13-O-345; and (**d**) 13-N-4, 13-N-34, and 13-N-345 in THF (1 × 10^−5^ mol·L^−1^).

The normalized PL spectra of the compounds in dilute THF solution (1 × 10^−5^ mol·L^−1^) are shown in Figure 3. These PL spectra are determined by the linking group and rigid structure, but are not affected by the alkyl tail chain. Both linear ester-type compounds have a *λ*_max_ located at 426 nm (Figure 3a). The same linear compounds but with the amide linking group instead have a *λ*_max_ of 460 nm (Figure 3b), indicating that the amide group strengthens the conjugated structure. Furthermore, the bent-shaped rigid structure reduces the conjugation of compounds. Compared to the linear compounds, the bent-shaped ester-type and amide-type compounds have *λ*_max_ values blue-shifted by around 30 nm, located at 400 nm (Figure 3c) and 430 nm (Figure 3d), respectively.

**Figure 4:**
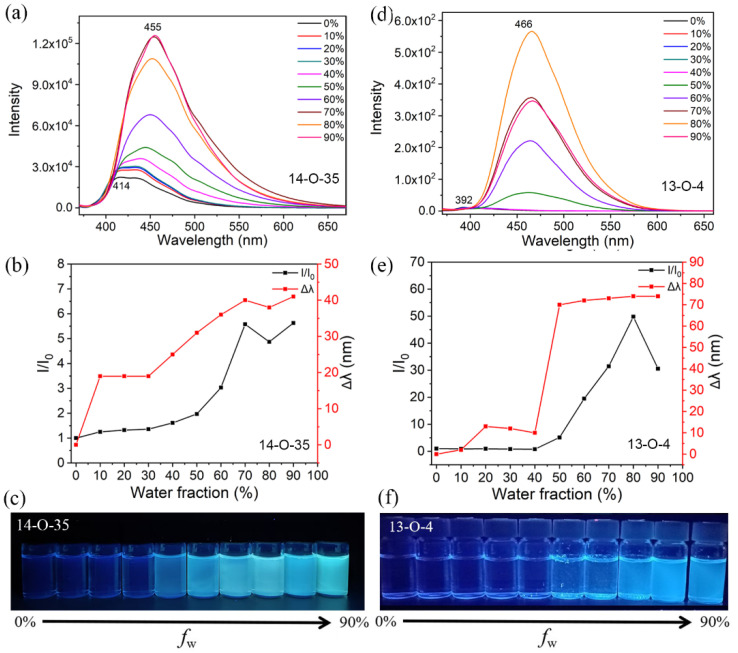
PL spectra of 14-O-35 (**a**) and 13-O-4 (**d**) in THF/water solution with different water fractions (*f*_w_); PL intensity and Δ*λ* of 14-O-35 (**b**) and 13-O-4 (**e**) as a function of *f*_w_ in THF/water mixtures; fluorescence pictures of 14-O-35 (**c**) and 13-O-4 (**f**) in THF/water solution with different *f*_w_ taken under 365 nm UV light.

The AIE activities of the compounds were investigated by studying their PL spectra changes in THF/water mixtures. As shown in Figure 4a,b, the ester-type compound 14-O-35 emits weakly in pure THF solution. The fluorescence intensity gradually increases when the water fraction (*f*_w_) is increased, and a sharp increase can be noticed when *f*_w_ reaches 50% (Figure 4b). The images in Figure 4c show that bluish-green fluorescence can be identified by the naked eye when *f*_w_ is 50% or higher. Meanwhile, the solution is no longer transparent as aggregates are formed. This is a typical aggregation-induced fluorescence enhancement phenomenon. Figure 4b also shows that *λ*_max_ red-shifts from 414 nm to 455 nm during aggregation, which is attributed to intramolecular planarization and the formation of *J*-type aggregates. 13-O-4 exhibits similar phenomena in THF/water solutions, as shown in Figure 4d. An increase in the PL intensity of 13-O-4 solutions also occurs when *f*_w_ is larger than 50% (Figure 4e). 13-O-4 in solution exhibits a 72 nm red-shift in *λ*_max_ from 392 nm to 466 nm during aggregation. Both compounds present the characteristic AIE effect (Figure 4c,f). The other ester-type compounds also exhibit AIE activities (Figure S3).

**Figure 5:**
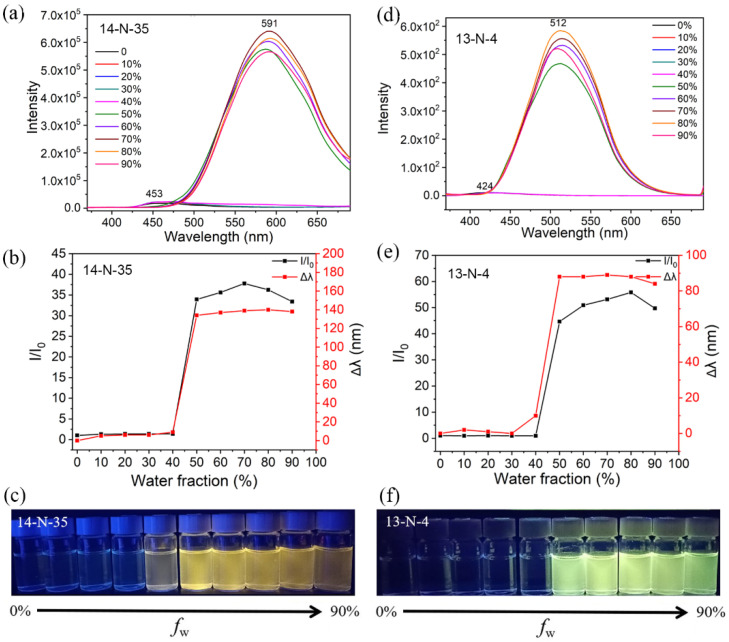
PL spectra of 14-N-35 (**a**) and 13-N-4 (**b**) in THF/water solution with different water fractions (*f*_w_); PL intensity and Δ*λ* of 14-N-35 (**c**) and 13-N-4 (**d**) as a function of *f*_w_ in THF/water mixtures; fluorescence pictures of 14-N-35 (**e**) and 13-N-4 (**f**) in THF/water solution with different *f*_w_ taken under 365 nm UV light.

The amide-type compounds under discussion also show AIE activities in THF/water mixtures, as shown in Figure 5 and Figure S4. The linear amide-type compounds in the THF/water mixtures show a more obvious red-shift in *λ*_max_ than the linear ester-type compounds. As shown in Figure 5a,c, the PL intensity of 14-N-35 is weak in pure THF. When the *f*_w_ reaches 50%, the PL intensity sharply increases, and *λ*_max_ moves from 453 nm to 591 nm, indicative of a large red-shift of about 138 nm. Meanwhile, the *λ*_max_ of 14-N-345 exhibits a 58 nm red-shift from 456 nm to 514 nm (Figure S4). During aggregation, the hydrogen bond between the amide-type compounds provides additional intermolecular interactions, resulting in a larger red-shift in emission than that of ester-type compounds. The bent-shaped amide-type compounds also exhibit AIE activities (Figure 5 and Figure S4). Similarly, the emission peak intensity of 13-N-4 dramatically increases when *f*_w_ reaches 50% (Figure 5b,e), with *λ*_max_ being red-shifted by approximately 90 nm. In short, most compounds experience a *λ*_max_ red-shift of varying degrees when *f*_w_ is raised from 0 to 90%. Due to the aggregation-induced large red-shift behavior, the *λ*_max_ of the linear and bent-shaped amide-type compounds aggregates reach at least 500 nm, exhibiting various emission colors of green and yellow (Figure 5c,f).

**Figure 6:**
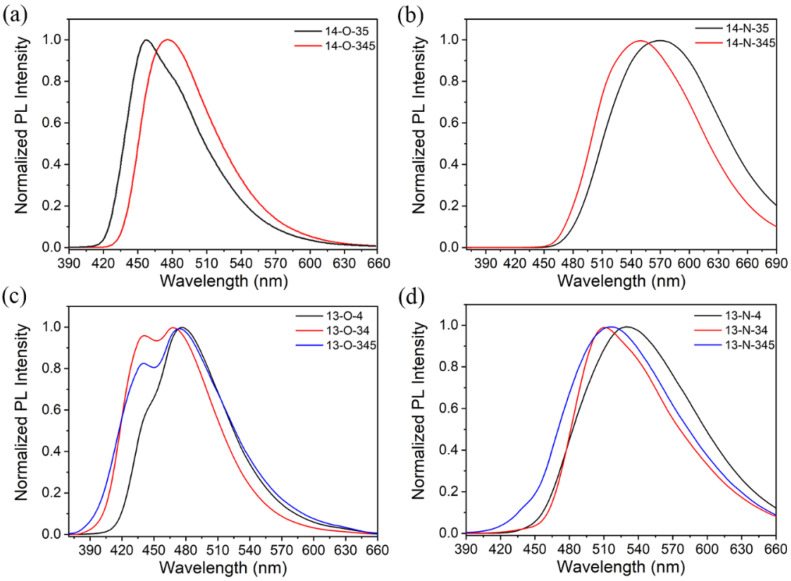
PL spectra of (**a**) 14-O-35 and 14-O-345; (**b**) 14-N-35 and 14-N-345; (**c**) 13-O-4, 13-O-34, and 13-O-345; and (**d**) 13-N-4, 13-N-34, and 13-N-345 in solid state.

Compared to the fluorescence properties of the compounds in solution, the PL spectra of the compounds in solid states show an obvious red-shift, with varying *λ*_max_ values. As shown in Figure 6a, the *λ*_max_ values of the emission peak for 14-O-35 and 14-O-345 are 460 nm and 480 nm, respectively. The *λ*_max_ of 14-N-35’s PL spectra is near 590 nm, while that of 14-N-345 is around 550 nm (Figure 6b), achieving over a 100 nm red-shift in the emission peak. The bent-shaped ester-type compounds, 13-O-4, 13-O-34, and 13-O-345, have a *λ*_max_ located at 480 nm (Figure 6c), close to that of the linear compounds but with multiple emission peaks, indicating their more complicated molecular arrangements in solid states. The DSC result of 13-O-4 in Table 1 shows its polycrystalline behavior. 13-O-345 has a crystallization temperature of around 44 °C, which is near room temperature. In addition, the bent-shaped ester-type compounds have the shortest effective conjugation length, which give rise to relatively weak intermolecular interactions among those four sets of compounds. Thus, their molecular arrangement would be more complicated than that of the other three sets of compounds.

The bent-shaped amide-type compounds have a *λ*_max_ around 510–540 nm (Figure 6d). In the solid state, the amide-type compounds, with their stronger intermolecular interactions, show a bigger red-shift in *λ*_max_ than the ester-type compounds. Different from the negligible alkoxy chain’s influence on the PL spectra in solution (Figure 3), variation in the maximum emission peak around 10~40 nm could be detected in each set of compounds (Figure 6), revealing that the soft aliphatic structure also has a slight effect on photophysical properties in a condensed state. However, the structural effect on the PL spectra is not consistent in these four sets of compounds. It is well known that both molecular structure and aggregates can alter photophysical properties in condensed states. It seems the structure of alkoxy tails does not directly affect the PL spectra like the conjugated part. The number and position of substituted alkoxy tails first affect molecular stacking, which, in turn, affect the PL spectra to some extent [68]. As shown in the CIE 1931chromaticity diagram (Figure S5), the emission color of those compounds in solid states changes from blue to yellow by altering the rigid core, linking groups, and alkoxy tails. Considering that molecular stacking, which is closely related to the emission color, can be adjusted by controlling the processing conditions, such as the temperature, solvent, mechanical force, and time, more detailed research on the influence of a condensed structure on fluorescence properties is ongoing.

### 2.4. Thermochromic and Photochromic Fluorescence Response

**Figure 7:**
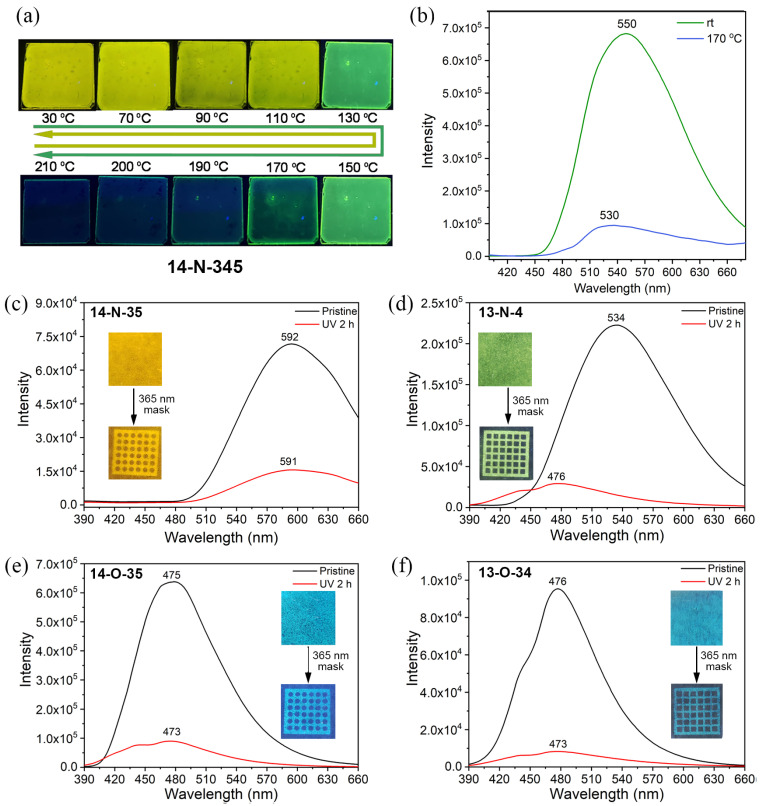
(**a**) Fluorescence images of 14-N-345 film taken under 365 nm UV light at indicated temperatures; (**b**) PL spectra of 14-N-345 at room temperature (rt) and 170 °C; PL spectra of 14-N-35 (**c**), 13-N-4 (**d**), 14-O-35 (**e**), and 13-O-34 (**f**) before and after irradiating for 2 h under 365 nm UV light (insets show fluorescence images under UV light before and after 365 nm irradiation through mask).

It has been found that amide-type compounds exhibit stronger red-shifted emission in solid state than in solution due to the hydrogen bonding interaction and π-π interaction. Their thermochromic fluorescence-responsive properties were investigated because the intermolecular interactions are also sensitive to temperature. The solution-casted 14-N-345 film shows a yellowish-green color at room temperature under 365 nm irradiation and temperature-dependent fluorescence variations (Figure 7a). As the temperature increases, the yellowish-green color changes rapidly to green when heated above 130 °C. After further heating above 170 °C, approaching an isotropic temperature of 14-N-345, the fluorescence intensity dramatically decreases. The emission change in the 14-N-345 film was further investigated using PL spectra (Figure 7b). The film shows an emission peak at 550 nm at room temperature. As the temperature increases to 170 °C, the emission peak is blue-shifted to 530 nm, accompanied by a substantial intensity decrease. The fluorescence color change is also reversible. The original emission is recovered when the film is cooled to room temperature, resulting in reversible thermochromic fluorescence switching. Other amide-type compounds also show similar thermochromic fluorescence responses.

A photochromic fluorescence response is also found in this system. Compounds were deposited on filter paper by spraying dichloromethane (DCM) solutions onto the paper and then dried up. Changes are visible in the PL spectra after irradiation under 365 nm UV light. For instance, a dramatic decrease in the emission intensity of the 14-N-35 film is observed, as shown in Figure 7c. Such a decrease is also confirmed by the *Φ*_F_ change. After irradiation, the *Φ*_F_ of the 14-N-35 film decreases from 35.8% to 9.5%. As a result, the high-contrast fluorescence pattern can easily be written in the 14-N-35 film when the pristine film is irradiated under 365 nm through a mask for 2 h (Figure 7c). The fluorescence intensity of the irradiated zone is much lower than that of the non-irradiated zone. Such a substantial decrease in intensity is attributed to the photoreaction occurring in the CS groups, which tends to decrease the conjugation of the rigid core. The bent-shaped amide-type compounds show the same intensity decrease after UV irradiation. For instance, the *Φ*_F_ of the 13-N-4 film decreases from 23.4% to 1.6% after irradiation. Furthermore, an emission peak blue-shift of around 58 nm can also be detected, as shown in Figure 7d. Because of the big difference in fluorescence intensity, only a high-contrast pattern can be observed instead of a two-color pattern, as shown in the inset of Figure 7d. The other bent-shaped amide-type compounds show similar photoresponsive behavior, resulting in a blue-shifted emission wavelength and reduced *Φ*_F_ (Figure S6). Compared to the ≥20 nm blue-shift in the amide-type compounds, all ester-type compounds show a fluorescence intensity decrease after irradiation but with only a ~5 nm blue-shift in the emission wavelength (Figure 7e,f). Thus, a blue fluorescence pattern can easily be written in the film under UV light through a mask. Since each compound contains two CS groups, the corresponding photochemical reaction is complicated. Even in solution, the photoreaction products include Z-E, E-E, and E-Z isomers. Meanwhile, the irradiated film becomes partially insoluble, which may be attributed to the existence of [2 + 2] cycloaddition. After irradiation, the written fluorescence pattern is quite stable when the film is kept in the dark.

## 3. Experimental Section

### 3.1. Materials and Methods

Methyl 4-hydroxybenzoate (99%), methyl gallate (99%), 1,4-benzenediacetonitrile (97%), hydroxybenzaldehyde (98%), and pyridinium dichromate (PDC) were purchased from Aladdin Industrial Co., Ltd., (Los Angeles, CA, USA). Methyl 3,4-dihydroxybenzoate (98%), methyl 3,5-dihydroxybenzoate (99%), methyl 3,5-dihydroxybenzoate (99%), and tetrabutylammonium hydroxide solution in MeOH (40%) were purchased from Energy Chemical Co., Ltd., (Shanghai, China). 1,3-benzenediacetonitrile (98%) and 4-aminobenzyl alcohol (98%) were purchased from Shanghai Macklin Biochemical Technology Co., Ltd. (Shanghai, China). DCM, THF, and n-dodecyl bromide (≥98%) were purchased from Shanghai Titan Scientific Co., Ltd. (Shanghai, China). The other reagents and solvents were purchased from Jiangsu Chinasun Specialty Products Co., Ltd., (Changshu, Jiangsu, China), and Sinopharm Chemical Reagent Co., Ltd., (Shanghai, China). Before use, DCM and THF were treated with sodium and then redistilled. All other reagents and solvents were used without further purification.

The ^1^H NMR spectra were recorded on a Bruker-New Advance NEO 300 MHz NMR spectrometer with CDCl_3_ or deuterated DMSO as the solvent and tetramethylsilane (TMS) as the internal standard. The mass spectrometry (MS) spectra of the photoproducts were recorded on a matrix-assisted laser desorption/ionization time-of-flight (MALDI-TOF) mass spectrometer (Ultra Extreme, Bruker Co., Leipzig, Germany).

The LC textures and birefringence of the samples were examined under a Leica DMRX POM (Leica Microsystems Inc., Morrisville NC, USA) equipped with a Linkam THMS 600 (Linkam Scientific Instrument Ltd., Salfords, UK) hot stage. The samples were made by sandwiching the compound powder between a glass slide and a cover glass. These samples were heated to their isotropic temperature and then cooled to room temperature at a rate of 1–5 °C/min. The thermal stability of all compounds was measured by TGA on TA Discovery TGA55 (TA Instruments, New Castle, DE, USA) instrument at a heating rate of 10 °C/min from room temperature to 800 °C under a nitrogen atmosphere. DSC thermograms were recorded under a nitrogen atmosphere by a TA DSC250 instrument (TA Instruments, USA). The temperature and heat flow were calibrated using standard materials (indium and zinc) at a cooling and heating rate of 10 °C/min. Samples with a typical mass of about 5 mg were encapsulated in sealed aluminum pans. The DSC curves recorded their first cooling and second heating processes at a rate of 10 °C/min. To identify the phase structures of compounds, SAXS experiments were performed using a high-flux X-ray instrument SAXSess mc2, (Anton Paar Co., Graz, Austria) equipped with a Kratky block-collimation system and a GEID3003 sealed-tube X-ray generator (Cu Kα). The wavelength was 0.1542 nm. The samples were wrapped in aluminum foil and sandwiched in a steel sample holder. The X-ray scattering patterns were recorded in a vacuum on an imaging plate (IP) which extended to a high-angle range (the *q* range covered from 0.06 to 29 nm^−1^). The diffraction peak positions were calibrated with silver behenate.

The UV-vis spectra were measured on a Shimadzu UV 1900i (Shimadzu Co., Tokyo, Japan). All samples in dilute organic solvents (1 × 10^−5^ mol·L^−1^) were prepared in the dark. The PL spectra were recorded on a Shimadzu RF-6000 spectrofluorophotometer (Shimadzu Co., Japan), with an excitation wavelength of 350 nm for solids and solutions. The fluorescence absolute values of ΦF were obtained on a Hamamatsu QY-Plus C13534-11 with an integrating sphere.

### 3.2. Synthesis of Compounds

The compounds were synthesized according to the synthetic route shown in Scheme S1.

#### 3.2.1. Synthesis of Linear and Bent-Shaped Ester Compounds

3,4,5-tris(dodecyloxy)benzoic acid (345-COOH): Methyl gallate (1.84 g, 10.00 mmol), anhydrous K_2_CO_3_ (10.10 g, 72.00 mmol), and 0.10 g KI were added to 100 mL acetone. The compound n-dodecyl bromide (8.90 g, 34.00 mmol) was added with stirring. The mixture was heated to reflux and checked via thin-layer chromatography (TLC) until the reaction was complete. The reaction mixture was poured into a suction filter to remove insoluble matter. The acetone was removed by rotary evaporation to give a solid. A mixture of 193 mL ethyl alcohol (EtOH) and 7 mL H_2_O was added to dissolve it. Under stirring, NaOH (2.47 g, 44.00 mmol) was added, and the mixture was heated under reflux and detected by TLC until the reaction was complete. Dilute acid was added to the reaction mixture to adjust the pH to acidic after cooling to RT. Then, it was poured into crushed ice and a white solid precipitate was obtained after suction filtration. After drying under vacuuming and recrystallization from EtOH, 9.12 g of 345-COOH pure product was obtained. Yield 86.6%. ^1^H NMR (300 MHz, CDCl_3_), δ: 7.34–7.32 (s, 2H), 4.10–3.98 (m, 6H), 1.87–1.72 (m, 6H), 1.60–1.42 (m, 6H), 1.40–1.21 (m, 48H), 0.90 (t, *J* = 6.0 Hz, 9H).

3,4,5-tris(dodecyloxy)benzoyl chloride (345-COCl): A mixture of 345-COOH (1.17 g, 1.74 mmol), thionyl chloride (SOCl_2_, 25 mL), and a drop of N, N’-dimethylformamide (DMF) was refluxed at 80 °C for 4 h. Thereafter, the SOCl_2_ was distilled off to produce solid 345-COCl.

(2Z,2′Z)-2,2′-(1,4-phenylene)bis(3-(4-hydroxyphenyl)acrylonitrile) (14-O): Hydroxybenzaldehyde (2.00 g, 16.40 mmol) and 1,4-benzenediacetonitrile (1.03 g, 6.60 mmol) were dissolved in 1-propanol (18 mL) and acetic acid (AcOH, 0.79 mL). The solution was heated to reflux under N_2_, and piperidine (0.97 mL) was slowly added. After 24 h, the reaction mixture was cooled to RT and the solid was filtered and washed with 1-propanol and then with methanol (MeOH). Yield 87.7%. ^1^H NMR (300 MHz, DMSO-*d*6), δ: 10.31 (s, 2H), 7.99 (s, 2H), 7.89 (d, *J* = 6.0 Hz, 4H), 7.82 (s, 4H), 6.93 (d, *J* = 6.0 Hz, 4H).

(2Z,2′Z)-2,2′-(1,3-phenylene)bis(3-(4-hydroxyphenyl)acrylonitrile) (13-O): The synthesis of 13-O was similar to that of 14-O, only replacing 1, 4-phenyldiacetonitrile with 1, 3-phenyldiacetonitrile. Yield: 85.3%. ^1^H NMR (300 MHz, DMSO-*d*6), δ: 10.47–10.1 (s, 2H), 8.00 (s, 2H), 7.98 (s, 1H), 7.90 (d, *J* = 6.0 Hz, 4H), 7.73 (dd, *J* = 7.2, 1.7 Hz, 2H), 7.61 (m, 1H), 6.94 (d, *J* = 9.0 Hz, 4H).

((1Z,1′Z)-1,4-phenylenebis(2-cyanoethene-2,1-diyl))bis(4,1-phenylene) bis(3,4,5-tris(dodecyloxy)benzoate) (14-O-345): The synthesis procedure of 14-O-345 was given as a representative example for ester compounds. A solution of 345-COCl (1.20 g, 1.74 mmol) in 20 mL DCM was added to a solution of 14-O (0.18 g, 0.50 mmol), N,N-diethylethanamine (TEA, 0.50 mL) in 30 mL DCM in an ice water bath and then stirred at RT overnight. The mixture was washed with saturated sodium bicarbonate aqueous solution and water. The solution was distilled off to produce a solid. The resulting solid was purified by silica gel column chromatography (DCM: petroleum ether = 1: 2) to yield a white solid of 0.69 g. Yield 82.2%. ^1^H NMR (300 MHz, CDCl_3_), δ: 8.04 (d, *J* = 9.0 Hz, 4H), 7.81 (s, 4H), 7.65 (s, 2H), 7.44 (s, 4H), 7.37 (d, *J* = 9.0 Hz, 4H), 4.14–4.02 (m, 12H), 1.93–1.72 (m, 12H), 1.55–1.43 (m, 12H), 1.40–1.24 (m, 96H), 0.90 (t, *J* = 6.0 Hz, 18H). MS (MALDI-TOF): Calculated for C_110_H_168_N_2_O_10_, *m*/*z*: 1677.270, *m*/*z* + Na+: 1700.260; Found, 1700.622. The other ester compounds were synthesized by a similar procedure to that used for 14-O-345.

((1Z,1′Z)-1,3-phenylenebis(2-cyanoethene-2,1-diyl))bis(4,1-phenylene)bis(3,4,5-tris(dodecyloxy)benzoate) (13-O-345): The synthesis of 13-O-345 was similar to that of 14-O-345, only replacing 14-O with 13-O. Yield: 81.6%. ^1^H NMR (300 MHz, CDCl_3_), δ: 8.02 (d, *J* = 8.6 Hz, 4H), 7.94 (s, 1H), 7.74 (dd, *J* = 7.8, 1.7 Hz, 2H), 7.63 (s, 2H), 7.61–7.53 (m, 1H), 7.41 (s, 4H), 7.35 (d, *J* = 6.0 Hz, 4H), 4.16–4.00 (m, 12H), 1.94–1.73 (m, 12H), 1.59–1.46 (m, 12H), 1.43–1.21 (m, 96H), 0.88 (t, *J* = 6.0 Hz, 18H). MS (MALDI-TOF): Calculated for C_110_H_168_N_2_O_10_, *m*/*z*: 1677.270, *m*/*z* + Na+: 1700.260; Found, 1700.605.

((1Z,1′Z)-1,4-phenylenebis(2-cyanoethene-2,1-diyl))bis(4,1-phenylene)bis(3,5-bis(dodecyloxy)benzoate) (14-O-35): Yield: 78.6%. ^1^H NMR (300 MHz, CDCl_3_), δ: 8.04 (d, *J* = 9.0 Hz, 4H), 7.81 (s, 4H), 7.64 (s, 2H), 7.38 (d, *J* = 9.0 Hz, 4H), 7.35 (d, *J* = 2.3 Hz, 4H), 6.75 (s, 2H), 4.11–3.99 (m, 8H), 1.92–1.76 (m, 8H), 1.54–1.45 (m, 8H), 1.44–1.20 (m, 64H), 0.91 (t, *J* = 6.0 Hz, 12H). MS (MALDI-TOF): Calculated for C_86_H_120_N_2_O_8_, *m*/*z*: 1308.904, *m*/*z* + Na+: 1331.894; Found, 1332.291.

((1Z,1′Z)-1,3-phenylenebis(2-cyanoethene-2,1-diyl))bis(4,1-phenylene)bis(4-(dodecyloxy)benzoate) (13-O-4): Yield: 83.2%. ^1^H NMR (300 MHz, CDCl_3_), δ: 8.18 (d, *J* = 9.0 Hz, 4H), 8.04 (d, *J* = 8.7 Hz, 4H), 7.96 (s, 1H), 7.75 (dd, *J* = 7.8, 1.7 Hz, 2H), 7.65 (s, 2H), 7.60–7.51 (m, 1H), 7.38 (d, *J* = 9.0 Hz, 4H), 7.02 (d, *J* = 9.0 Hz, 4H), 4.14–4.01 (m, 4H), 1.93–1.78 (m, 4H), 1.54–1.44 (m, 4H), 1.43–1.17 (m, 32H), 0.90 (t, *J* = 6.0 Hz, 6H). MS (MALDI-TOF): Calculated for C_62_H_72_N_2_O_6_, *m*/*z:* 940.539, *m*/*z* + Na+: 963.529; Found, 963.849.

((1*Z*,1′*Z*)-1,3-phenylenebis(2-cyanoethene-2,1-diyl))bis(4,1-phenylene)bis(3,4-bis(dodecyloxy)benzoate) (13-O-34): Yield: 81.2%. ^1^H NMR (300 MHz, CDCl_3_, TMS), δ (ppm): 8.04 (d, *J* = 9.0 Hz, 4H), 7.96 (s, 1H), 7.86 (dd, *J* = 8.4, 2.1 Hz, 2H), 7.76 (dd, *J* = 7.8, 1.8 Hz, 2H), 7.69 (d, *J* = 2.0 Hz, 2H), 7.65 (s, 2H), 7.62–7.54 (m, 1H), 7.38 (d, *J* = 9.0 Hz, 4H), 6.97 (d, *J* = 8.4 Hz, 2H), 4.17–4.06 (m, 8H), 1.95–1.81 (m, 8H), 1.56–1.46 (m, 8H), 1.45–1.21 (m, 64H), 0.90 (t, *J* = 6.0 Hz, 12H). MS (MALDI-TOF): Calculated for C_86_H_120_N_2_O_8_, *m*/*z*: 1308.904, *m*/*z* + Na+: 1331.894; Found, 1332.270.

#### 3.2.2. Synthesis of Linear and Bent-Shaped Amide Compounds

3,4,5-tris(dodecyloxy)-N-(4-(hydroxymethyl)phenyl)benzamide (HO-NH-345): To a mixture of 4-aminobenzyl alcohol (0.12 g, 1 mmol) in 10 mL THF, 0.5 mL TEA was added. After stirring for 30 min, 345-COCl (0.83 g, 1.2 mmol) was added at RT. The reaction mixture was stirred at RT for 5 h. The reaction mixture was poured into water and the pH adjusted to about 6, and then, the mixture was extracted with ethyl acetate 3 times. The combined organic layers were dried over MgSO_4_, filtered, and concentrated in vacuo. The solid was purified by silica gel column chromatography (DCM: ethyl acetate = 8: 1) to produce 0.65 g HO-NH-345. Yield 83.3%. ^1^H NMR (300 MHz, CDCl_3_), δ (ppm): 7.71 (s, 1H), 7.64 (d, *J* = 9.0 Hz, 2H), 7.40 (d, *J* = 9.0 Hz, 2H), 7.07 (s, 2H), 4.70 (s, 2H), 4.09–3.97 (m, 6H), 1.83–1.57 (m, 6H), 1.56–1.39 (m, 6H), 1.39–1.41 (m, 48H), 0.90 (t, *J* = 6.0 Hz, 9H).

3,4,5-tris(dodecyloxy)-N-(4-formylphenyl)benzamide) (NH-345): PDC (1.45 g, 3.85 mmol), 15 drops of AcOH, and 2.25 g kieselguhr were added to a mixture of HO-NH-345 (1.50 g, 1.92 mmol) in 90.00 mL DCM. The mixture was stirred at RT for 24 h under a nitrogen atmosphere. Then, to the mixture was added enough diethyl ether. The mixture was dried over MgSO4, filtered, and concentrated in vacuo. The solid was purified by silica gel column chromatography (DCM: ethyl acetate = 20: 1) to produce 1.30 g NH-345. Yield 86.9%. ^1^H NMR (300 MHz, CDCl_3_), δ (ppm): 9.98 (s, 1H), 7.98–7.87 (m, 3H), 7.85 (d, *J* = 8.6 Hz, 2H), 7.07 (s, 2H), 4.10–3.98 (m, 6H), 1.88–1.68 (m, 6H), 1.52–1.38 (m, 6H), 1.39–1.00 (m, 48H), 0.90 (t, *J* = 6.0 Hz, 12H).

*N*,*N*’-(((1Z,1′Z)-1,4-phenylenebis(2-cyanoethene-2,1-diyl))bis(4,1-phenylene))bis(3,4,5-tris(dodecyloxy)benzamide) (14-N-345): The synthesis procedure of 14-N-345 was given as a representative example for amide compounds. An amount of 0.50 mL tetrabutylammonium hydroxide solution in MeOH (40%) was added to a solution of NH-345 (0.25 g, 0.32 mmol) and 1,4-benzenediacetonitrile (0.02 g, 0.15 mmol) in 25 mL EtOH at 55 °C. After the monitoring of the reaction by TLC was completed, the reaction mixture was cooled to RT. A yellow solid was collected through simple filtration. The resulting solid was purified by silica gel column chromatography (DCM: petroleum ether = 1: 1) to yield a luminous yellow solid of 0.18 g. Yield 73.5%. ^1^H NMR (300 MHz, CDCl_3_), δ (ppm): 8.01 (d, *J* = 8.5 Hz, 4H), 7.91 (s, 2H), 7.81 (d, *J* = 8.9 Hz, 4H), 7.78 (s, 4H), 7.60 (s, 2H), 7.09 (s, 4H), 4.14–3.99 (m, 12H), 1.92–1.70 (m, 12H), 1.57–1.43 (m, 12H), 1.44–1.19 (m, 96H), 0.91 (t, *J* = 6.0 Hz, 18H). MS (MALDI-TOF): Calculated for C_110_H_170_N_4_O_8_, *m*/*z*: 1676.305, *m*/*z* + Na+: 1699.295; Found, 1699.049. The other amide compounds were synthesized by a similar procedure to that used for 14-N-345.

*N*,*N*’-(((1Z,1′Z)-1,3-phenylenebis(2-cyanoethene-2,1-diyl))bis(4,1-phenylene))bis(3,4,5-tris(dodecyloxy)benzamide) (13-N-345): The synthesis of 13-N-345 was similar to that of 14-N-345, only replacing 1, 4-phenyldiacetonitrile with 1, 3-phenyldiacetonitrile. ^1^H NMR (300 MHz, CDCl_3_), δ (ppm): 8.01 (d, J = 8.6 Hz, 4H), 7.94 (s, 1H), 7.89 (s, 2H), 7.81 (d, *J* = 8.7 Hz, 4H), 7.72 (dd, *J* = 6.0, 1.7 Hz, 2H), 7.60 (s, 2H), 7.60–7.57 (m, 1H), 7.09 (s, 4H), 4.11–3.97 (m, 12H), 1.91–1.70 (m, 12H), 1.52–1.40(m, 12H), 1.41–1.07 (m, 96H), 0.90 (t, *J* = 6.0 Hz, 18H). MS (MALDI-TOF): Calculated for C_110_H_170_N_4_O_8_, *m*/*z*: 1676.305, *m*/*z* + Na+: 1699.295; Found, 1699.070.

*N*,*N*’-(((1Z,1′Z)-1,4-phenylenebis(2-cyanoethene-2,1-diyl))bis(4,1-phenylene))bis(3,5-bis(dodecyloxy)benzamide) (14-N-35): Yield: 74.3%. ^1^H NMR (300 MHz, CDCl_3_), δ (ppm): 7.98 (d, J = 8.7 Hz, 4H), 7.96 (s, 2H), 7.79 (d, J = 8.8 Hz, 4H), 7.75 (s, 4H), 7.57 (s, 2H), 6.97 (d, *J* = 2.2 Hz, 4H), 6.63 (s, 2H), 4.06–3.94 (m, 8H), 1.86–1.74 (m, 8H), 1.52–1.41 (m, 8H), 1.39–1.19 (m, 64H), 0.87 (t, *J* = 6.0 Hz, 12H). MS (MALDI-TOF): Calculated for C_86_H_122_N_4_O_6_, *m*/*z*: 1306.936, *m*/*z* + Na+: 1329.926; Found, 1330.214.

*N*,*N*’-(((1Z,1′Z)-1,3-phenylenebis(2-cyanoethene-2,1-diyl))bis(4,1-phenylene))bis(4-(dodecyloxy)benzamide) (13-N-4): Yield: 68.5%. ^1^H NMR (300 MHz, CDCl_3_), δ (ppm): 8.01 (d, *J* = 8.5 Hz, 4H), 7.93 (s, 1H), 7.92–7.88 (m, 6H), 7.82 (d, *J* = 8.6 Hz, 4H), 7.72 (dd, *J* = 6.0, 1.7 Hz, 2H), 7.60 (s, 2H), 7.58–7.50 (m, 1H), 7.01 (d, *J* = 8.6 Hz, 4H), 4.09–4.02 (m, 4H), 1.90–1.78 (m, 4H), 1.54–1.44 (m, 4H), 1.42–1.20 (m, 32H), 0.91 (t, *J* = 6.0 Hz, 6H). MS (MALDI-TOF): Calculated for C_62_H_74_N_4_O_4_, *m*/*z*: 938.571, *m*/*z* + Na+: 961.561; Found, 961.863.

*N*,*N*’-(((1*Z*,1′*Z*)-1,3-phenylenebis(2-cyanoethene-2,1-diyl))bis(4,1-phenylene))bis(3,4-bis(dodecyloxy)benzamide) (13-N-34): Yield: 71.3%. ^1^H NMR (300 MHz, CDCl_3_), δ (ppm): 8.04–7.96 (m, 6H), 7.93 (s, 1H), 7.82 (d, *J* = 8.5 Hz, 4H), 7.72 (dd, *J* = 6.0, 1.7 Hz, 2H*),* 7.60 (s, 2H), 7.59–7.54 (m, 1H), 7.52 (d, *J* = 2.1 Hz, 2H), 7.42 (dd, *J* = 8.5, 2.2 Hz, 2H), 6.95 (d, *J* = 8.4 Hz, 2H), 4.15–4.03 (m, 8H), 1.93–1.81 (m, 8H), 1.60–1.44 (m, 8H), 1.44–1.20 (m, 64H), 0.89 (t, *J* = 6.0 Hz, 12H). MS (MALDI-TOF): Calculated for C_86_H_122_N_4_O_6_, *m*/*z*: 1306.936, *m*/*z* + Na+: 1329.926; Found, 1330.373.

## 4. Conclusions

In this study, we designed and synthesized a series of CS-based compounds. Their LC phase behaviors and photophysical properties related to their molecular structural parameters, such as the shape of the rigid core, the linking groups, and the number of substituted alkoxy tails, were investigated. It was found that bent-shaped compounds are more prone to exhibiting thermotropic LC phases than linear compounds. Compounds with one alkoxy tail exhibit SmC structures; bent-shaped compounds with multi-tail chain substitutions possess Col_h_ structures. All the compounds exhibit AIE properties in THF/water mixtures. When *f*_w_ increases to a certain threshold, a dramatic emission intensity increase and an emission wavelength red-shift are detected. The maximum emission peaks of the ester-type compounds in solid states are around 480 nm, and those of the amide-type compounds are extended to 590 nm, exhibiting tunable fluorescence colors. This is the first time the wavelength of the emission peak range has been extended to more than 100 nm, just by changing the linking group from an ester to an amide group. The amide-type compounds also show reversible thermochromic fluorescence switching and exhibit a photochromic fluorescence response with high-contrast fluorescence patterns. Further investigation of stimulus-induced fluorescence response properties is still ongoing. This work provides a new strategy for the design and synthesis of fluorescent liquid crystalline materials with multiple responsive properties for various optoelectronic applications.

## Figures and Tables

**Table 1:** Phase transition temperatures of compounds and corresponding enthalpies.

Compound	T/°C [∆H/kJ·mol^−1^)]
**14-O-35**	Cr 80 [20.69] Iso (Iso 74 [13.29] Cr)
**14-O-3** **4** **5**	Cr 112 [20.78] Iso (Iso 103 [21.07] Cr)
**14-N-35**	Cr 210 [34.88] Iso (Iso 200 [36.04] Cr)
**14-N-345**	Cr 185 [28.19] Iso (Iso 180 [4.01] M174 [24.01] Cr)
**13-O-4**	Cr_1_ 113 [12.54] Cr_2_ 135 [21.08] SmC 165 [18.07] Iso (Iso 163 [17.01] SmC 100 [11.94] Cr)
**13-O-34**	Cr 107 [34.87] Col_h_ 115 ^#^ Iso (Iso 110 ^#^ Col_h_ 91 [48.91] Cr)
**13-O-345**	Cr 44 [12.56] Col_h_ 65 [2.30] Iso (Iso 60 [2.43] Col_h_ 40 [10.75] Cr)
**13-N-4**	Cr 165 SmC 238 [25.21] Iso (Iso 228 [25.42] SmC 160 ^#^ Cr)
**13-N-34**	Cr 121 Col_h_ 164 [0.81] Iso (Iso 160 ^#^ Col_h_ 112 [1.96] Cr)
**13-N-345**	Col_h_ 125 [1.14] Iso (Iso 115 [0.94] Col_h_)

Cr = crystal; SmC = smectic C phase; Col_h_ = hexagonal columnar phase; M = unknown mesophase; Iso = isotropic liquid; ^#^: data were obtained from POM.

## Data Availability

The data presented in this study are available in the Supplementary Materials.

## Supplementary Materials

The following supporting information can be downloaded at: https://www.mdpi.com/article/10.3390/molecules29235811/s1, Scheme S1: synthesis routes of compounds; Figure S1: POM pictures of 14-N-345; Figure S2: UV-Vis absorption spectra of compounds in THF; Figures S3–S5: PL spectra in different states. Figure S6: PL spectra of 14-O-345(a), 14-N-345(b), 13-N-34(c), 13-N-345 (d) before and after irradiating for 2 h under 365 nm UV light.

## References

[1] Wang Y.F., Shi J.W., Chen J.H., Zhu W.G., Baranoff E. (2015). Recent progress in luminescent liquid crystal materials: Design, properties and application for linearly polarised emission. J. Mater. Chem. C.

[2] Uchida J., Soberats B., Gupta M., Kato T. (2022). Advanced functional liquid crystals. Adv. Mater..

[3] Kato T., Uchida J., Ichikawa T., Sakamoto T. (2018). Functional liquid crystals towards the next generation of materials. Angew. Chem. Int. Edit..

[4] Yamane S., Tanabe K., Sagara Y., Kato T., Tschierske C. (2012). Stimuli-responsive photoluminescent liquid crystals. Liquid Crystals: Materials Design and Self-Assembly.

[5] Bisoyi H.K., Li Q. (2022). Liquid crystals: Versatile self-organized smart soft materials. Chem. Rev..

[6] Li X., Yang H., Zheng P., Lin D.M., Zhang Z.J., Kang M.M., Wang D., Tang B.Z. (2023). Aggregation-induced emission materials: A platform for diverse energy transformation and applications. J. Mater. Chem. A.

[7] Zhao Z., Zhang H., Lam J.W.Y., Tang B.Z. (2020). Aggregation-induced emission: New vistas at the aggregate level. Angew. Chem. Int. Ed..

[8] Zhang H.K., Zhao Z., Turley A.T., Wang L., McGonigal P.R., Tu Y.J., Li Y.Y., Wang Z.Y., Kwok R.T.K., Lam J.W.Y. (2020). Aggregate science: From structures to properties. Adv. Mater..

[9] Mei J., Leung N.L.C., Kwok R.T.K., Lam J.W.Y., Tang B.Z. (2015). Aggregation-Induced Emission: Together we shine, united we soar!. Chem. Rev..

[10] Hong Y., Lam J.W.Y., Tang B.Z. (2011). Aggregation-induced emission. Chem. Soc. Rev..

[11] An B.K., Kwon S.K., Jung S.D., Park S.Y. (2002). Enhanced emission and its switching in fluorescent organic nanoparticles. J. Am. Chem. Soc..

[12] An B.K., Gierschner J., Park S.Y. (2012). π-Conjugated cyanostilbene derivatives: A unique self-assembly motif for molecular nanostructures with enhanced emission and transport. Acc. Chem. Res..

[13] Zhu L.L., Zhao Y.L. (2013). Cyanostilbene-based intelligent organic optoelectronic materials. J. Mater. Chem. C.

[14] Gierschner J., Park S.Y. (2013). Luminescent distyrylbenzenes: Tailoring molecular structure and crystalline morphology. J. Mater. Chem. C.

[15] Martinez-Abadia M., Gimenez R., Ros M.B. (2018). Self-assembled α-cyanostilbenes for advanced functional materials. Adv. Mater..

[16] Lin S.Y., Gutierrez-Cuevas K.G., Zhang X.F., Guo J.B., Li Q. (2021). Fluorescent photochromic α-cyanodiarylethene molecular switches: An emerging and promising class of functional diarylethene. Adv. Funct. Mater..

[17] Mahalingavelar P., Kanvah S. (2022). α-Cyanostilbene: A multifunctional spectral engineering motif. Phys. Chem. Chem. Phys..

[18] Afrin A., Swamy P.C.A. (2024). Symphony of light: AIE and MFC in carbazole-based cyanostilbenes. J. Mater. Chem. C.

[19] He X., Wei P. (2024). Recent advances in tunable solid-state emission based on α-cyanodiarylethenes: From molecular packing regulation to functional development. Chem. Soc. Rev..

[20] Shen Y.H., Yao K., Li H., Xu Z.R., Quan Y.W., Cheng Y.X. (2022). Strong CPL-active liquid crystal materials induced by intermolecular hydrogen-bonding interaction and a chirality induction mechanism. Soft Matter.

[21] Jiang Q., Ruan H., Sun C.C., Wang T., Zhang Y.P., Qiu Y., Wang H., Liao Y.G., Xie X.L. (2023). Two-color cholesteric liquid crystalline gels for reversible writing and erasing information encryption. Macromol. Rapid Comm..

[22] Kunzelman J., Kinami M., Crenshaw B.R., Protasiewicz J.D., Weder C. (2008). Oligo(p-phenylene vinylene)s as a “New” class of piezochromic fluorophores. Adv. Mater..

[23] Aoki H., Mihara T., Koide N. (2004). Synthesis and thermal properties of twin dimers containing cyanostilbene groups as a mesogenic group. Mol. Cryst. Liq. Cryst..

[24] Fang Z.M., Zou K.S., Wu C.C. (2020). Novel cholesterol-based liquid crystalline dimers: The α-cyanostilbene group served as a photofluorophore and mesogenic moiety. Liq. Cryst..

[25] Bao R., Pan M., Qiu J.J., Liu C.M. (2010). Synthesis and characterization of six-arm star-shaped liquid crystalline cyclotriphosphazenes. Chin. Chem. Lett..

[26] Martinez-Abadia M., Varghese S., Milian-Medina B., Gierschner J., Gimenez R., Blanca Ros M. (2015). Bent-core liquid crystalline cyanostilbenes: Fluorescence switching and thermochromism. Phys. Chem. Chem. Phys..

[27] Martinez-Abadia M., Robles-Hernandez B., Villacampa B., de la Fuente M.R., Gimenez R., Ros M.B. (2015). Cyanostilbene bent-core molecules: A route to functional materials. J. Mater. Chem. C.

[28] Martinez-Abadia M., Robles-Hernandez B., de la Fuente M.R., Gimenez R., Ros M.B. (2016). Photoresponsive cyanostilbene bent-core liquid crystals as new materials with light-driven modulated polarization. Adv. Mater..

[29] Cao X.J., Li W., Li J.H., Zou L., Liu X.W., Ren X.K., Yu Z.Q. (2022). Controlling the balance of photoluminescence and photothermal effect in cyanostilbene-based luminescent liquid crystals. Chin. J. Chem..

[30] Liang Y.R., Liu X.T., Hu X.N., Li X.H., Liu N.N., Xiao Y.L. (2024). Terminal halogen-containing rod-like liquid crystals: Synthesis, self-assembly, photophysical and mechanochromism properties. Spectrochim. Acta A.

[31] Liu Z.G., Liu X.T., Shen B.Y., Liang Y.R., Zhang S.B., Hu X.N., Zhang R.L., Xiao Y.L. (2024). The influence of positional isomerism of a cyano group of pyrene-based rod-like mesogens on mesomorphism, dual-state emission and multiple applications. Tetrahedron.

[32] Lu H.B., Zhang S.N., Ding A.X., Yuan M., Zhang G.Y., Xu W., Zhang G.B., Wang X.H., Qiu L.Z., Yang J.X. (2014). A luminescent liquid crystal with multistimuli tunable emission colors based on different molecular packing structures. New J. Chem..

[33] Mihara T., Ito M., Koide N. (2004). Synthesis and thermal properties of twin dimers containing cyanostilbene derivatives as a mesogenic group. Mol. Cryst. Liq. Cryst..

[34] Martinez-Abadia M., Varghese S., Gimenez R., Ros M.B. (2016). Multiresponsive luminescent dicyanodistyrylbenzenes and their photochemistry in solution and in bulk. J. Mater. Chem. C.

[35] Koo J., Jang J., Oh M., Kim M., Hyeong J., Lim S.I., Cao Y., Tran D.T., Park S., Jeong K.U. (2022). Cyanostilbene-based AIEgen smart film: Optical switching by engineering molecular packing structure and molecular conformation through thermal and photoinduced monotropic phase transition. Adv. Opt. Mater..

[36] Yoon S.J., Kim J.H., Kim K.S., Chung J.W., Heinrich B., Mathevet F., Kim P., Donnio B., Attias A.J., Kim D. (2012). Mesomorphic organization and thermochromic luminescence of dicyanodistyrylbenzene-based phasmidic molecular disks: Uniaxially aligned hexagonal columnar liquid crystals at room temperature with enhanced fluorescence emission and semiconductivity. Adv. Funct. Mater..

[37] Park J.W., Nagano S., Yoon S.J., Dohi T., Seo J., Seki T., Park S.Y. (2014). High contrast fluorescence patterning in cyanostilbene-based crystalline thin films: Crystallization-induced mass flow via a photo-triggered phase transition. Adv. Mater..

[38] Mu B., Zhao Y., Li X., Quan X.H., Tian W. (2020). Enhanced conductivity and thermochromic luminescence in hydrogen bond-stabilized columnar liquid crystals. ACS Appl. Mater. Interfaces.

[39] Zhang D.L., Liu Y.T., Gao H.F., Chang Q., Cheng X.H. (2020). α-Cyanostilbene and fluorene based bolaamphiphiles: Synthesis, self-assembly, and AIEE properties with potential as white-light emissive materials and light-emitting liquid crystal displays. J. Mater. Chem. C.

[40] Mu B., Zhang Z.L., Quan X.H., Hao X.N., Tian W. (2021). Perylene bisimide-based luminescent liquid crystals with tunable solid-State light emission. ACS Appl. Mater. Interfaces.

[41] Pang Y.D., Xiao Y.L., Liu X.T., Zuo R.Z., Li N.N., Jiang Z.L. (2021). Synthesis and characterization of α-cyanostilbene-based bent-core hexacatenar mesogens with different central groups. Tetrahedron.

[42] Wang R.Q., Chen S.B., Chen Q.R., Guo H.Y., Yang F.F. (2021). Porphyrin with circularly polarized luminescence in aggregated states. Dyes Pigment..

[43] Xiao Y.L., Liu X.T., He Q.Y., He X., Zhang D.X. (2020). Synthesis and self-assembly of benzothiadiazole-cored α-cyanostilbene hexacatenar mesogens. Soft Mater..

[44] Zuo R.Z., Wang S.W., Pang Y.D., Xiao Y.L., Jiang Z.L. (2021). Synthesis and characterization of AIE-active and photo-active α-cyanostilbene-containing carbazole-based hexacatenars. Dyes Pigment..

[45] Liu X.T., Zuo R.Z., Li N.N., Pang Y.D., Jiang Z.L., Xiao Y.L. (2021). First biscyanovinyl diphenylfluorenone-based hexacatenars: Synthesis, liquid crystal and photophysical properties. Phase Transit..

[46] Xiao Y.L., Liu X.T., Li N.N., Pang Y.D., Zheng Z. (2022). Central condensed ring changes for manipulating the self-assembly and photophysical behaviors of cyanostilbene-based hexacatenars. J. Mol. Liq..

[47] Chen Y.Q., Li Y.C., Liang Y.R., Liu X.T., Xiao Y.L. (2023). The effect of the number of cyanostilbene building blocks on the self-assembly and photophysical property of pyrene-based liquid crystals. Tetrahedron.

[48] Bala I., Kaur H., Maity M., Yadav R.A.K., De J., Gupta S.P., Jou J.H., Pandey U.K., Pal S.K. (2022). Electroluminescent aggregation-induced emission-active discotic liquid crystals based on alkoxy cyanostilbene-functionalized benzenetricarboxamide with ambipolar charge transport. ACS Appl. Electron. Mater..

[49] Zhao H.M., Cheng X.H. (2023). Fluorene thiophene α-cyanostilbene hexacatenar-generating LCs with hexagonal columnar phases and gels with helical morphologies as well as a light-emitting LC display. Int. J. Mol. Sci..

[50] Feng H.Y., He Y., Yang W.Q., Wang S., Feng Y.S. (2023). A novel strategy for constructing fluorescent liquid crystals with diphenylacrylonitrile groups derivatives based on thiazolo 5,4-d thiazole core. J. Mol. Struct..

[51] Gao T.Z., Liang Y.R., Liu N.N., Wen X.R., Liu X.T., Gao H.F., Xiao Y.L. (2023). The influence of positional isomerism of terminal alkyl chains on mesomorphic and photophysical behavior of unsymmetric α-cyanostilbene-based tetracatenars. J. Fluoresc..

[52] Liang Y.R., Gao T.Z., Hu X.N., Liu N.N., Liu X.T., Gao H.F., Xiao Y.L. (2024). The effect of fluorination on the liquid crystal and optical behaviors of amphiphilic cyanostilbene-based mesogens. J. Mol. Struct..

[53] Ding W., Chen S.B., Du X.Y., Cheng X.H. (2022). A self-assembled aza-BODIPY linked dicyanostilbenzene with a large Stokes shift, AIE, mechanochromism and singlet oxygen yield. J. Mol. Liq..

[54] Liu X.T., Li N.N., Pang Y.D., Li S.H., Xiao Y.L. (2022). Synthesis, self-assembly and photophysical properties of AIEE-active triphenylamine-based discotic liquid crystals. J. Iran. Chem. Soc..

[55] Lin L.B., Guo H.Y., Fanga X.T., Yang F.F. (2017). Novel AIE columnar liquid crystals: The influence of the number of diphenylacrylonitrile groups on the mesomorphic and fluorescence properties. Rsc Adv..

[56] Gupta R.K., Pathak S.K., De J., Pal S.K., Achalkumar A.S. (2018). Room temperature columnar liquid crystalline self-assembly of acidochromic, luminescent, star-shaped molecules with cyanovinylene chromophores. J. Mater. Chem. C.

[57] Chen S.B., Jiang S.J., Qiu J.B., Guo H.Y., Yang F.F. (2020). Dual-responding circularly polarized luminescence based on mechanically and thermally induced assemblies of cyano-distyrylbenzene hydrogen bonding liquid crystals. Chem. Commun..

[58] Zeng C.Y., Hu P., Wang B.Q., Fang W.Y., Zhao K.Q. (2023). Cyanostilbene bridged triphenylene dyad stimuli-responsive discotic liquid crystal: Synthesis, properties and applications. Chin. J. Org. Chem..

[59] Liang Y.R., Liu X.T., Pu Y., Zhang R.L., Zhou L.M., Xiao Y.L. (2023). Synthesis, LC self-assembly and photophysical behavior of stimuli responsive cyanostilbene-based hexacatenar mesogens with multicolour switching. J. Lumin..

[60] Ren Y.M., Zhang R.L., Yan C., Wang T.Y., Cheng H.F., Cheng X.H. (2017). Self-assembly, AIEE and mechanochromic properties of amphiphilic α-cyanostilbene derivatives. Tetrahedron.

[61] Zhao J., Chi Z.H., Zhang Y., Mao Z., Yang Z.Y., Ubba E., Chi Z.G. (2018). Recent progress in the mechanofluorochromism of cyanoethylene derivatives with aggregation-induced emission. J. Mater. Chem. C.

[62] Chung J.W., You Y., Huh H.S., An B.K., Yoon S.J., Kim S.H., Lee S.W., Park S.Y. (2009). Shear- and UV-induced fluorescence switching in stilbenic π-dimer crystals powered by reversible [2+2] cycloaddition. J. Am. Chem. Soc..

[63] Ping X.N., Pan J.J., Peng X., Yao C.Y., Li T., Feng H., Qian Z.S. (2023). Recent advances in photoresponsive fluorescent materials based on [2+2] photocycloaddition reactions. J. Mater. Chem. C.

[64] Huang Y.J., Ning L.J., Zhang X.M., Zhou Q., Gong Q.Y., Zhang Q.C. (2024). Stimuli-fluorochromic smart organic materials. Chem. Soc. Rev..

[65] Li P.Y., Wang J.X., Li P.F., Lai L.M., Yin M.Z. (2021). Minor alkyl modifications for manipulating the fluorescence and photomechanical properties in molecular crystals. Mater. Chem. Front..

[66] Wang Y., Shang H., Li B., Jiang S. (2022). Reversible luminescence “off-on” regulation based on tunable photodimerization via crystal-to-cocrystal transformation. J. Mater. Chem. C.

[67] Mu B., Zhang Z.L., Hao X.N., Ma T.S., Tian W. (2022). Positional Isomerism-Mediated Copolymerization Realizing the Continuous Luminescence Color-Tuning of Liquid-Crystalline Polymers. Macromolecules.

[68] Davis R., Kumar N.S.S., Abraham S., Suresh C.H., Rath N.P., Tamaoki N., Das S. (2008). Molecular packing and solid-state fluorescence of alkoxy-cyano substituted diphenylbutadienes: Structure of the luminescent aggregates. J. Phys. Chem. C.

