# Supplementary material for: Aggregation-Induced Emission-Active Cyanostilbene-Based Liquid Crystals: Self-Assembly, Photophysical Property, and Multiresponsive Behavior
Source: Molecules. 2024 Dec 9;29(23):5811. doi: 10.3390/molecules29235811 (PMC11643938; doi:10.3390/molecules29235811)
Supplement: Supplementary file 1 [file molecules-29-05811-s001.zip › molecules-3325291-supplementary.pdf]

# Supplementary information

## Aggregation-Induced Emission-Active Cyanostilbene-Based Liquid Crystals: Self-Assembly, Photophysical Property, and Multiresponsive Behavior

Bian Li, Junde Zhang, Juan Wang and Xiaofang Chen *

Suzhou Key Laboratory of Macromolecular Design and Precision Synthesis, Jiangsu Key Laboratory of Advanced Functional Polymer Design and Application, State and Local Joint Engineering Laboratory for Novel Functional Polymeric Materials, College of Chemistry, Chemical Engineering and Materials Science, Soochow University, Suzhou 215123, China

*Correspondence author: xfchen75@suda.edu.cn

## Content

1. Scheme S1. Synthetic routes for linear and bent-core compounds.
2. Figure S1. POM pictures of 14-N-345 taken at 188 °C (a) and 186 °C (b), when it was cooling from isotropic state.
3. Figure S2. UV-vis absorption spectra of (a) 14-O-35 and 14-O-345; (b) 14-N-35 and 14-N-345; (c) 13-O-4, 13-O-34 and 13-O-345; (d) 13-N-4, 13-N-34 and 13-N-345 in THF ($1\times10^{-5}$ mol $L^{-1}$).
4. Figure S3. PL spectra of (a) 14-O-345; (c) 13-O-4 in THF/water mixtures with different water fractions; Fluorescence pictures of (b) 14-O-345 and (d) 13-O-4 in THF/water with different water fractions ($f_w$) taken under 365 nm UV light.
5. Figure S4. PL spectra of (a) 14-N-345; (c) 13-N-34; (e) 13-N-345 in THF/water mixtures with different water fractions; Fluorescence pictures of (b)14-N-345; (d) 13-N-34 and (d) 13-N-345 in THF/water with different water fractions ($f_w$) taken under 365 nm UV light.
6. Figure S5. CIE chromaticity diagram of these compounds in solid state.
7. Figure S6. PL spectra of 14-O-345(a), 14-N-345(b), 13-N-34(c), 13-N-345 (d) before and after irradiating for 2 h under 365 nm UV light.

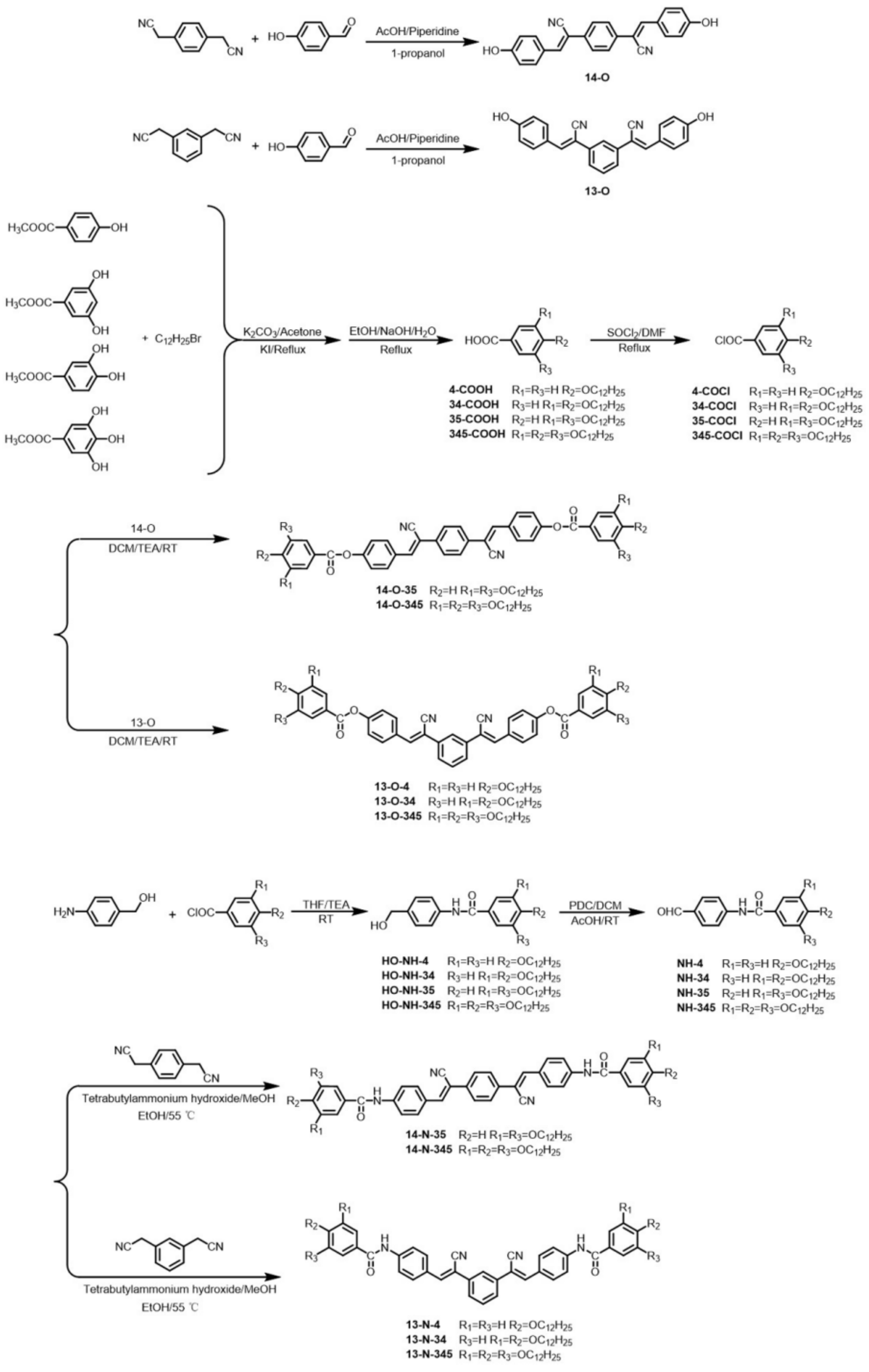


**Scheme S1.** Synthetic routes for linear and bent-core compounds.

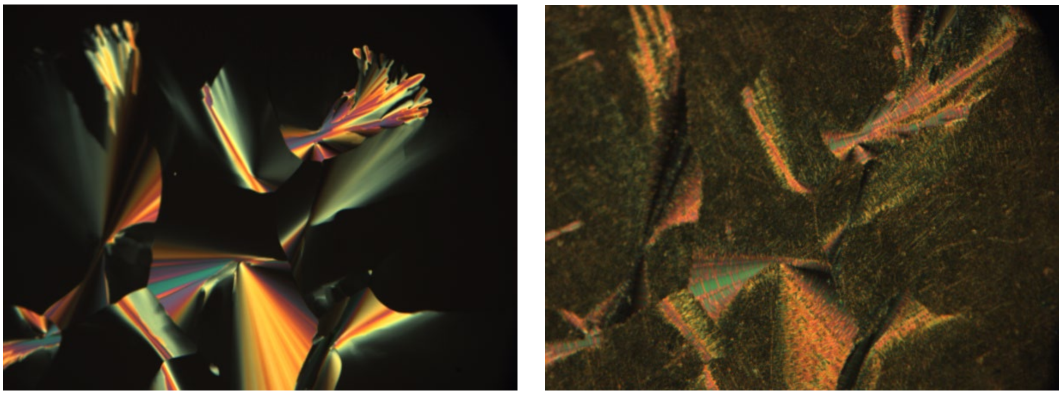

(a) (b)

**Figure S1.** POM pictures of 14-N-345 taken at 180 °C (a) and 176 °C (b), when it was cooling from isotropic state.

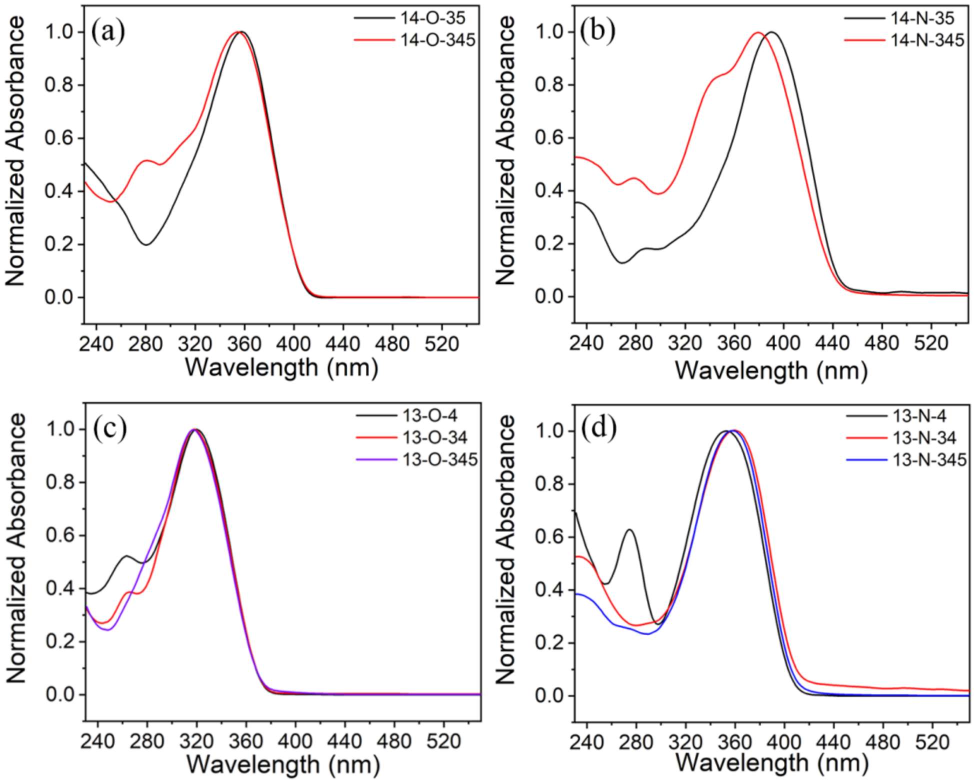


**Figure S2.** UV-vis absorption spectra of (a) 14-O-35 and 14-O-345; (b) 14-N-35 and 14-N-345; (c) 13-O-4, 13-O-34 and 13-O-345; (d) 13-N-4, 13-N-34 and 13-N-345 in THF ($1\times10^{-5}$ mol $L^{-1}$).

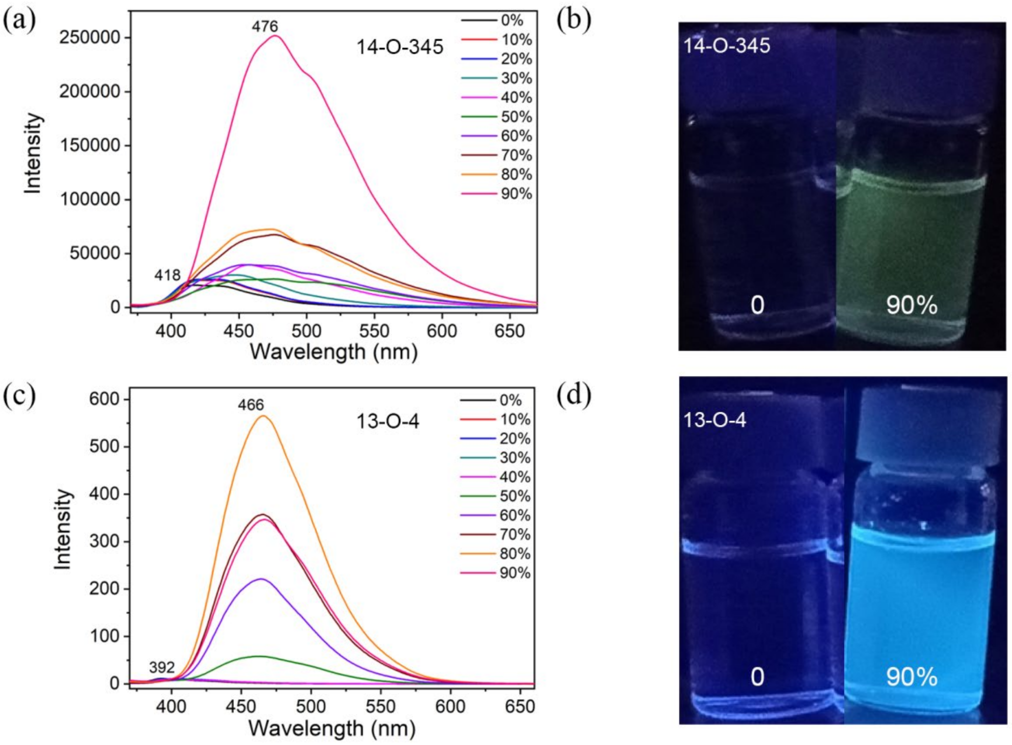


**Figure S3.** PL spectra of (a) 14-O-345; (c) 13-O-4 in THF/water mixtures with different water fractions; Fluorescence pictures of (b) 14-O-345 and (d) 13-O-4 in THF/water with different water fractions ($f_w$) taken under 365 nm UV light.

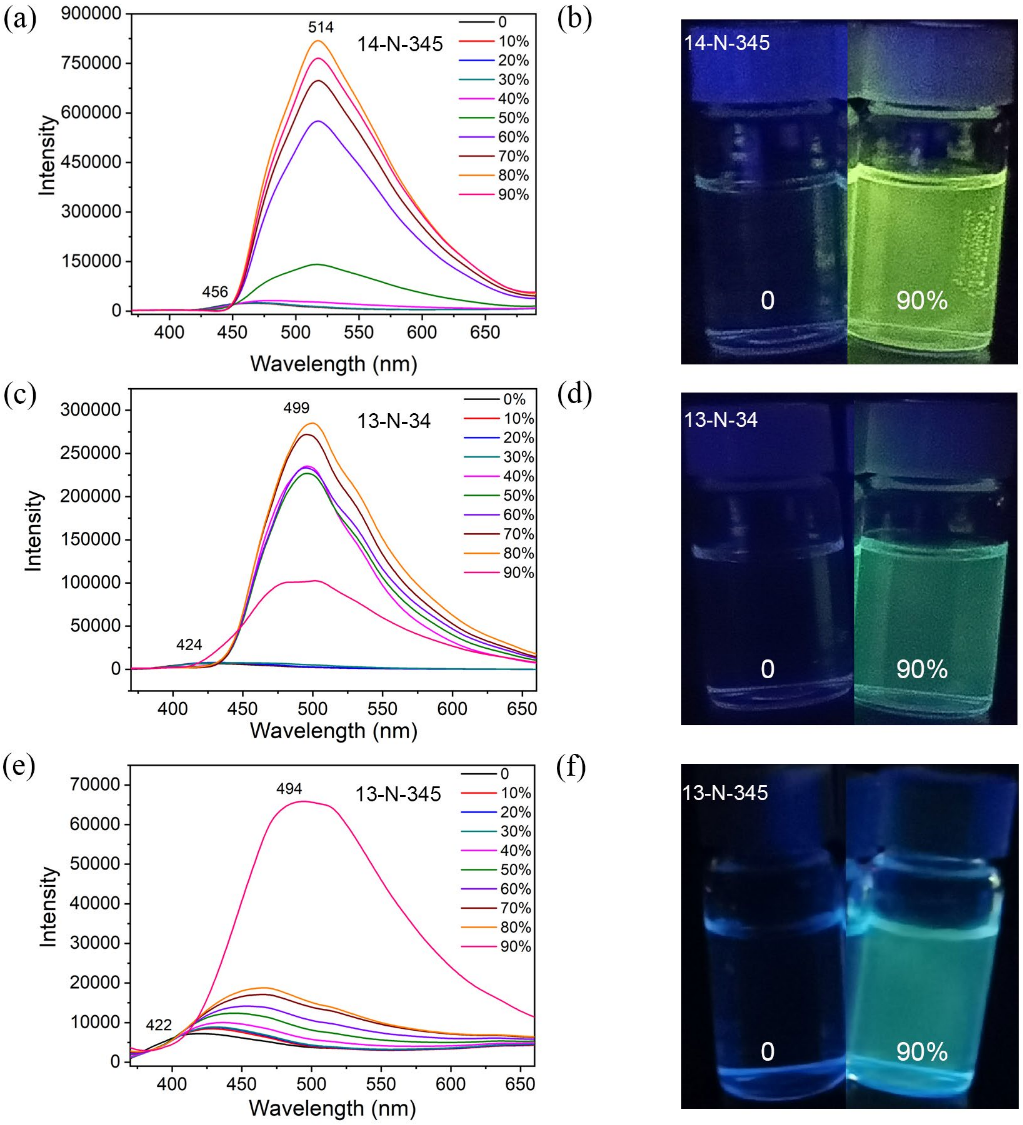


**Figure S4.** PL spectra of (a) 14-N-345; (c) 13-N-34; (e) 13-N-345 in THF/water mixtures with different water fractions; Fluorescence pictures of (b)14-N-345; (d) 13-N-34 and (d) 13-N-345 in THF/water with different water fractions ($f_w$) taken under 365 nm UV light.

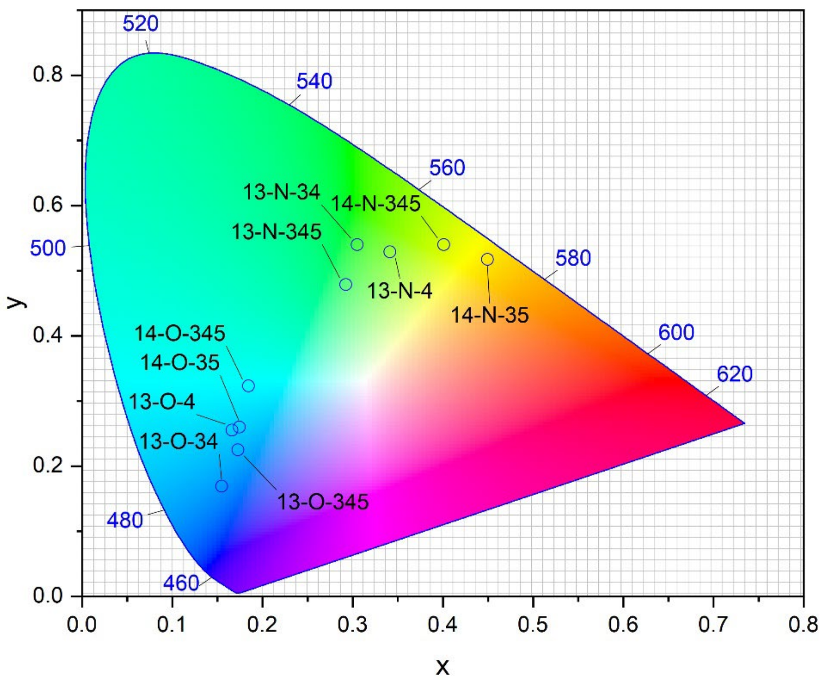


**Figure S5.** CIE chromaticity diagram of these compounds in solid state.

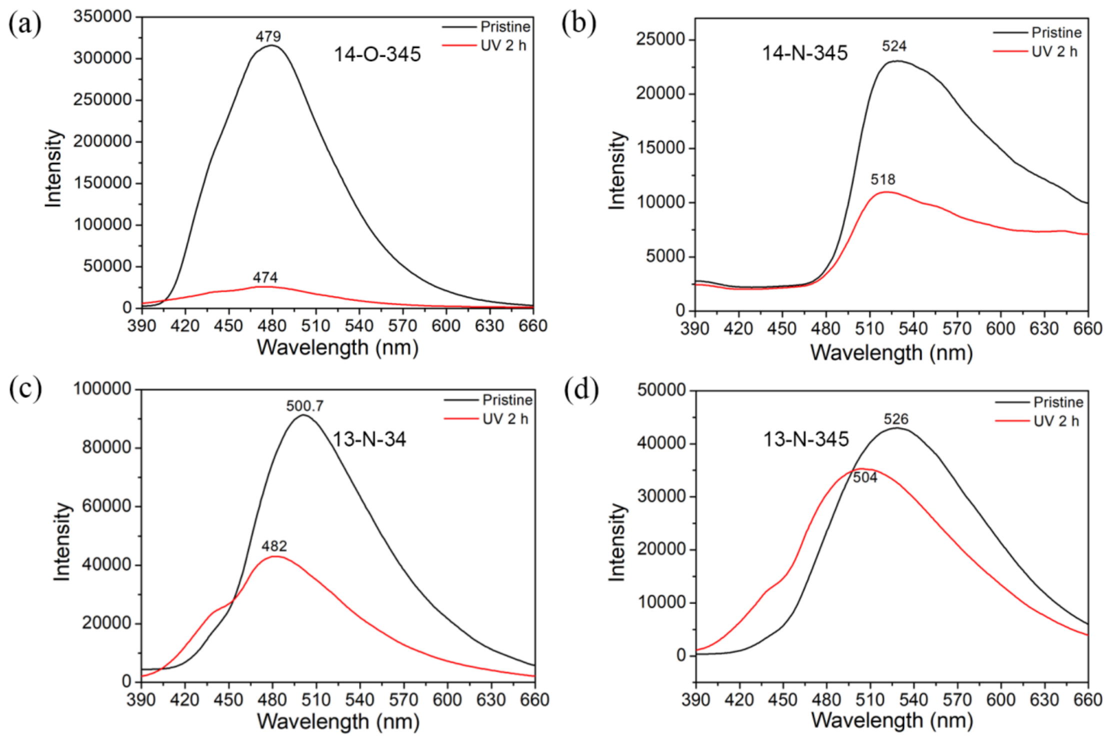


**Figure S6.** PL spectra of 14-O-345(a), 14-N-345(b), 13-N-34(c), 13-N-345 (d) before and after irradiating for 2 h under 365 nm UV light.
